# The clinical phenotype of carriers of intermediate alleles in the huntingtin gene: A scoping review

**DOI:** 10.1177/18796397251397683

**Published:** 2025-12-17

**Authors:** Anna van Hofslot, Mayke Oosterloo, Joost J.A. de Jong, Ruben L. Andriessen, Susanne T. de Bot, David E. J. Linden

**Affiliations:** 1 Mental Health and Neuroscience Research Institute (MHeNS), Faculty of Health, 82246 Medicine, and Life Sciences, 5211 Maastricht University, Maastricht, The Netherlands; 2 Department of Neurology, 5211 Maastricht University Medical Centre, Maastricht, The Netherlands; 3 Department of Neurology, 4501 Leiden University Medical Centre, Leiden, The Netherlands; 4 Department of Radiology & Nuclear Medicine, 199236 Maastricht University Medical Center, Maastricht, The Netherlands; 5 Department of Psychiatry, 5211 Maastricht University Medical Centre, Maastricht, The Netherlands

**Keywords:** intermediate alleles, intermediate repeat, IA carrier, CAG repeat, repeat expansion, huntington's disease, huntingtin gene, *HTT* gene

## Abstract

**Background:**

Huntington's Disease (HD) is a hereditary neurodegenerative disorder caused by a cytosine-adenine-guanine (CAG) repeat expansion (CAG > 35) in the Huntingtin (*HTT*) gene. Intermediate alleles (IAs, CAG = 27–35) are generally not associated with HD. However, IA carriers with symptoms have been reported in literature.

**Objective:**

To review the existing literature on IAs, in order to provide an overview of the clinical phenotype of IA carriers.

**Methods:**

Peer-reviewed articles published between 1993 and July 2024 from three databases (Embase, PubMed, and Web of Science) were included.

**Results:**

In case reports, a high percentage (90%) of IA carriers was reported to have symptoms (HD-related and -unrelated), or abnormalities in neuroimaging. Cohort studies also reported evidence of symptoms in IA carriers, although most cohorts did not obtain significant differences compared to controls.

**Conclusion:**

Based on this review, we argue that there is not enough evidence to draw a clear conclusion on the clinical phenotype of individuals carrying an intermediate allele of the *HTT* gene. Literature reports symptomatic IA carriers, but reported symptoms are non-specific and common in the general population. Additionally, the quality of the data is suboptimal, due to lack of detailed symptom descriptions, the absence of differential diagnoses, a selection bias, and a considerable publication bias towards IA carriers with symptoms. More research is needed to provide a better insight into the clinical phenotype of IA carriers.

## Introduction

Huntington's Disease (HD) is an autosomal dominant neurodegenerative disorder, caused by an abnormal expansion of the cytosine-adenine-guanine (CAG) repeat on the Huntingtin gene located on the short arm of chromosome 4.^1,2^ HD is characterized by involuntary movements, neuropsychiatric symptoms and cognitive deterioration. It has a variable age of onset, with an average age of onset of 30–50 years, and a known range of 2 to 85 years.^3^

**Figure 1.**
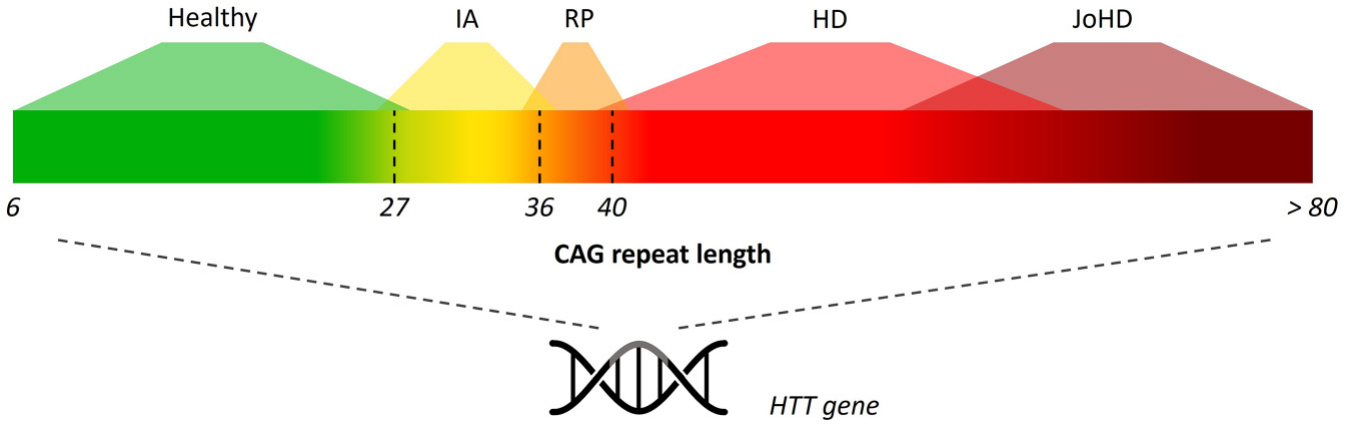
Spectrum of CAG repeat length in the huntingtin (*HTT*) gene. IA = intermediate allele, RP = reduced penetrance, HD = Huntington's disease, JoHD = Juvenile-onset Huntington's disease.

Repeat lengths vary in the population, in healthy individuals as well as in HD patients. Therefore, repeat length can be seen as a spectrum [Figure 1]. Patients with HD generally have a repeat length of ≥ 36 repeats, with repeat lengths of 36 up to and including 39 repeats associated with reduced penetrance.^3–7^ Repeat length is strongly associated with age of onset, and HD patients with a lower repeat length tend to have a later onset of the disease.^8–12^

In healthy individuals, the repeat length usually lies between 6 to 35 repeats.^5^ Alleles with a repeat length of 27 up to and including 35 are called intermediate alleles (IAs).^13,14^ These IA are known to show instability and have the potential to expand into the disease range within one or more generations.^13^ Although IAs lie within the healthy range and are not considered to be associated with HD, there have been reports of subjects with a repeat length of 27 to 35 who developed symptoms that are also seen in HD, such as chorea, depression, and cognitive decline.^5,7,15,16^ IA carriers can be identified through three types of testing: diagnostic testing for individuals with symptoms, predictive testing for those with a positive family history of HD, and testing for scientific research. An overview of all studies with IA carriers with and without symptoms is lacking so far, and there is currently no consensus in terms of the clinical phenotype of carriers of IAs.

To this end, we provide an overview of the clinical phenotype of IA carriers, by reviewing the existing literature on intermediate CAG repeat length in the *HTT* gene.

## Methods

This scoping review was conducted following the PRISMA-ScR (Preferred Reporting Items for Systematic reviews and Meta-Analysis for Scoping Reviews) guidelines, and provides a descriptive overview of existing literature.^17^

### Inclusion and exclusion criteria

For this review, we excluded articles that were published before 1993 [Table 1]. In 1993 the CAG repeat in the *HTT* was discovered as the cause of HD.^1^ Additionally, we excluded articles that focused on genetic counseling of persons with an intermediate repeat, and articles that were solely about CAG repeat instability over generations (in sperm or egg cells). All exclusion criteria are listed in Table 1.

**Table 1.** Inclusion and exclusion criteria.

Inclusion Criteria	Exclusion Criteria
The article contains information about intermediate alleles (defined as 27–35 repeats) in the *HTT* gene.	Review article.
In the article, the intermediate allele is the longer of the two alleles of the carrier(s).	The reference is a comment, correction, or reply to an article.
The article discusses neurological disease related clinical outcomes in IA carrier(s).	Not published in Dutch or English.
The study was done in humans.	No full text article available (conference abstracts, abstracts of poster presentations, etc.)
	Year of publishing before 1993.
	Articles solely about genetic counseling, or CAG instability over generations (in sperm or egg cells)

### Literature search strategy

The literature search strategy included key words “Huntington's Disease” and “Intermediate Alleles”, and their derivatives, using Embase, PubMed, and Web of Science, and was verified by a librarian of the University of Maastricht, The Netherlands (for full search strategy see Appendix 1). We included articles published from 1993 till July 2024. In addition, we manually searched the reference lists of articles included in the review for additional references.

### Screening

Duplicates were detected using Endnote (EndNote software, version 20.6,^18^) and Rayyan (https://www.rayyan.ai,^19^) as well as searched for manually, and were manually removed after verification by the first author (AvH). Two reviewers (AvH and RA) independently screened the titles and abstracts of the articles for in- and exclusion criteria [Table 1]. All articles included by at least one reviewer, were included in the second screening round. The second screening included reading the full article to reassess inclusion/exclusion. Results were compared and any differences were resolved by discussion and consulting the full text until consensus was reached. If consensus could not be reached, a third reviewer was consulted (MO).

### Data charting process cases

Data of individual IA carriers were extracted from case reports, case series, and cohort studies. IA carriers with symptoms resembling HD who had alternative genetic disorders confirmed by DNA analysis were excluded from this review. Demographic data is reported in this review, including sex, CAG repeat length of longer and shorter allele, and age at symptom onset. Additionally, we reported clinical data, such as symptoms, family history, medication use, and neuroimaging data. In this review we did not report post-mortem findings.

#### Nomenclature

In this review we use the term HGECs (Huntingtin gene expansion carrier, CAG ≥ 36), non-expanded (CAG < 36), IA carriers (CAG = 27–35 inclusive), and healthy controls (HCs, CAG < 27).

#### Symptom analysis

For the symptom analysis, we excluded IA carriers without clinical description. Additionally, we excluded IA carriers with a reported diagnosis (non-genetic) that provided an explanation for their symptoms. Symptoms were divided in motor, cognitive and neuropsychiatric symptoms. Neuropsychiatric symptoms were classified using the items used in the short version of the ‘Problem Behavior Assessment’ (PBA-s). The PBA-s is a shorter version of the PBA, specifically designed for use in HD.^20^ It consists of items evaluating 11 domains of psychological/behavioral signs and symptoms: depressed mood, suicidal ideation, anxiety, irritability, aggression, apathy, perseveration, obsessive-compulsive behavior, delusions, hallucinations, and confusion/disorientation.

The relationship between CAG repeat length and age at symptom onset was assessed using linear regression, with a significance level α of 0.05.

## Results

### Literature search results and screening

**Figure 2.**
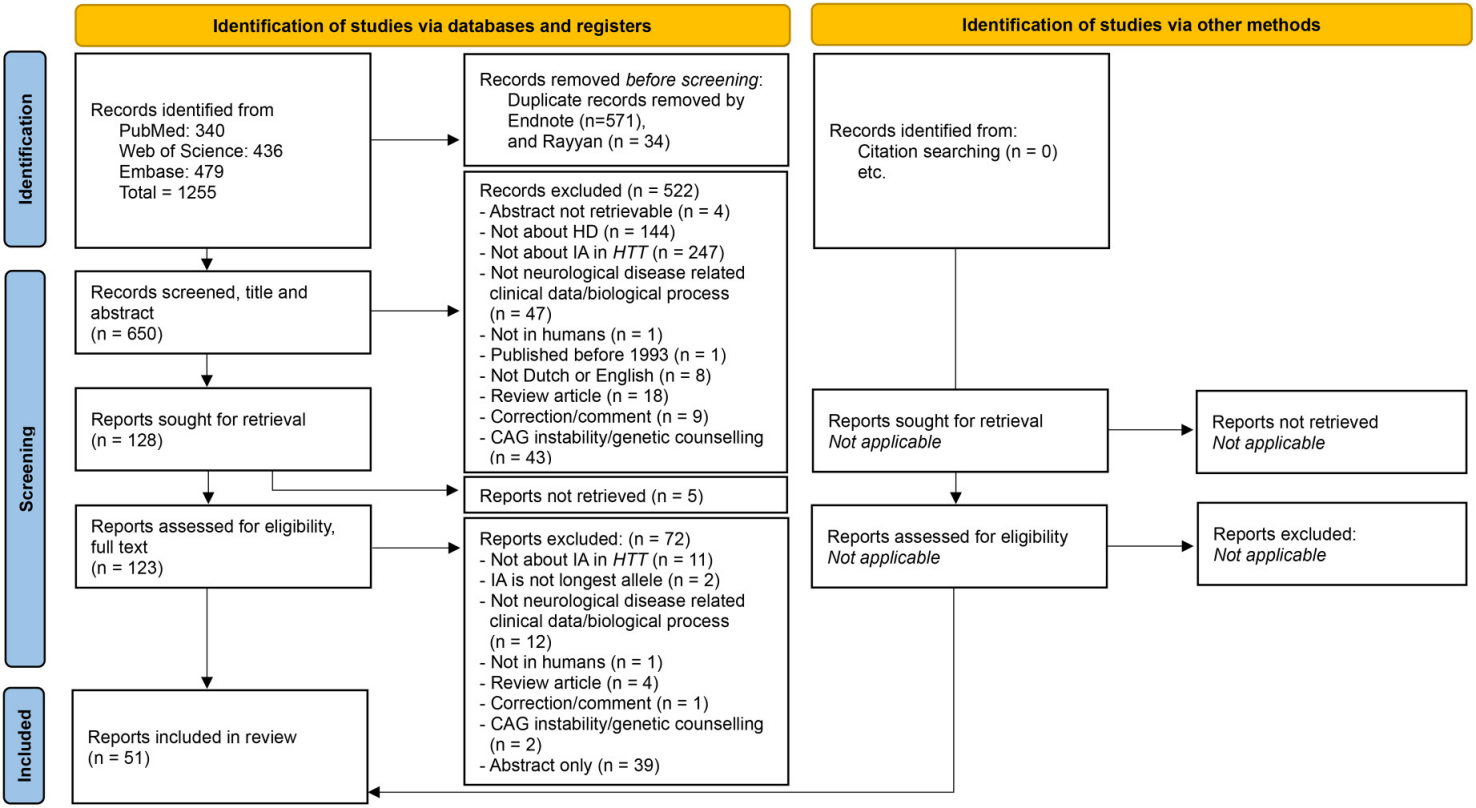
Flow chart of inclusion/exclusion of articles. *Flowchart from:* Page MJ, McKenzie JE, Bossuyt PM, Boutron I, Hoffmann TC, Mulrow CD, et al. The PRISMA 2020 statement: an updated guideline for reporting systematic reviews. BMJ 2021;372:n71. doi: 10.1136/bmj.n71.

The search strategy resulted in a total of 1255 articles, of which 650 articles remained after removal of duplicates. After two rounds of screening, 51 articles were included in this review. Reasons for exclusion were recorded [Figure 2].

### Data acquisition

**Figure 3.**
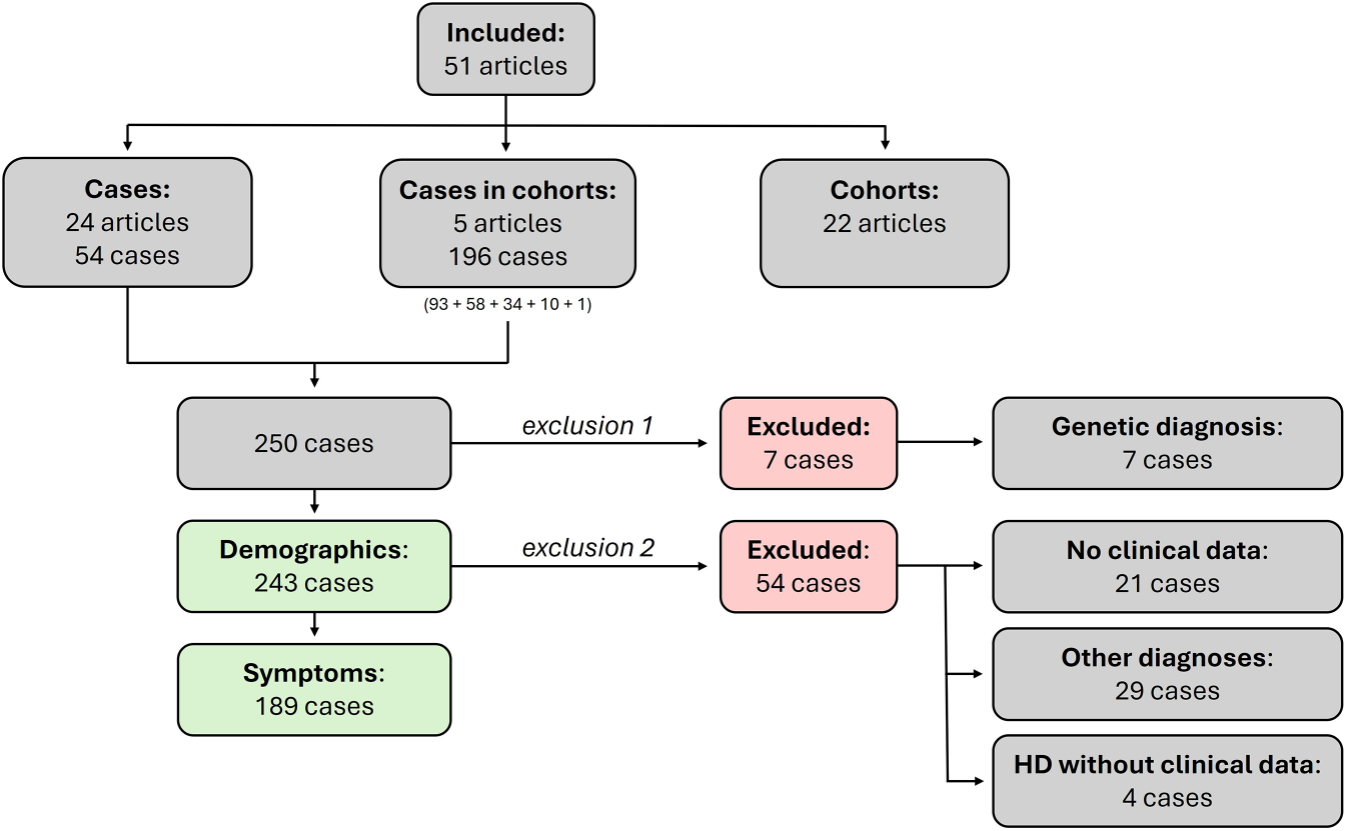
Flow chart of inclusion/exclusion of cases.

Out of the 51 included articles, 29 articles, of which 5 articles describing a cohort,^16,21–24^ reported a total of 250 IA carriers [Figure 3].^15,25–46^ We excluded seven cases from this review (exclusion 1), because they had a genetically confirmed diagnosis -besides their IA *HTT*- that could explain their symptoms and signs [Supplemental Table 1]. The remaining 243 IA carriers were included in this review [Figure 3].

Additionally, 22 articles investigated a cohort of IA carriers, or a cohort of otherwise defined subjects containing one or more IA carriers.^47–68^ IA cases and cohorts were described separately in this review.

### Cases

#### Demographics

A total of 243 IA carriers were included in this review, of whom 111 were male, 100 were female, and in 32 of the IA carriers the sex remained unknown. The CAG repeat length of the longer allele had a mean of 29.6 repeats (n = 243, SD = 2.3). The CAG repeat length of the shorter allele was reported in 232 of the IA carriers (95.5%), with a mean of 18.5 repeats (SD = 3.1).

#### Symptoms

For the data analysis of the reported symptoms in IA carriers, a selection was made to ensure meaningful quantification [Figure 3]. A total of 21 IA carriers were excluded from the symptom analysis, because no clinical data were available [Supplemental Table 2].^16,21,24,28,46^ Additionally, 29 IA carriers were excluded from the symptom analysis, because their reported diagnoses provided a full or nearly complete explanation for their symptoms. These IA carriers are listed in Table 2. Thirteen IA carriers (6.8% of all IA carriers) had a clinical HD diagnosis. However, four of these had no further symptom descriptions and were therefore excluded from the main symptom analysis [Supplemental Table 3].^7,27,29,31–34^^,43^ Thus, a total of 54 IA carriers were excluded from the symptom analysis, and 189 IA carriers remained. Out of 189, 19 (10.0%) IA carriers were reported to be healthy or asymptomatic [Supplemental Table 4].^16,23,35,46^ The other 170 IA carrier were reported to have at least one type of symptom.

**Table 2.** IA carriers that were excluded from symptom analysis.

Diagnosis	Number of cases (n)	Symptom description available (yes/no)	Case number	References
**Cases with symptom description**				
Parkinson's disease or Parkinsonism	13	Yes	Case 37, 45, 61, 89	^21^
		Yes	Case 8, 9	^25^
		Yes	Case 1, 26	^16^
		Yes	Case report	^37^
		Yes	Case report	^41^
		Yes	Case 4	^38^
		Yes	Case report	^44^
		Yes	Case 17	^23^
Sydenham's chorea	1	Yes	Case 2	^30^
Progressive supranuclear palsy (PSP)	1	Yes	Case 2	^40^
Multiple stroke induced chorea	1	Yes	Case 19	^16^
Alzheimer's disease	2	Yes	Case 46	^16^
		Yes	Case report	^45^
Conversion disorder	1	Yes	Case 27	^16^
Brain concussion with coma	1	Yes	Case 3	^16^
Symptoms after stroke	1	Yes	Case 47	^16^
**Total:**	21			
**Cases without symptom description**				
Conversion disorder	4	No	Case 6, 17, 25, 43	^16^
Mitochondrial encephalopathy	1	No	Case 2	^16^
Juvenile myoclonus epilepsy	1	No	Case 23	^16^
**Total:**	6			
**Cases with drug or alcohol abuse**				
Cognitive impairment due to alcohol abuse	1	No	Case 38	^16^
Chorea due to alcohol abuse	1	No	Case 45	^16^
**Total:**	2			
**Overall total:**	29			

**Figure 4.**
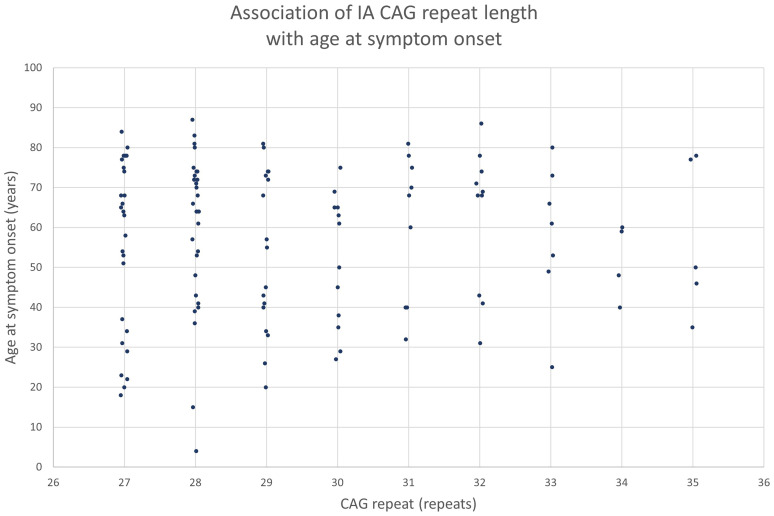
Graph of the association of IA CAG repeat length with age at symptom onset. CAG = cytosine, adenine, guanine.

**Figure 5.**
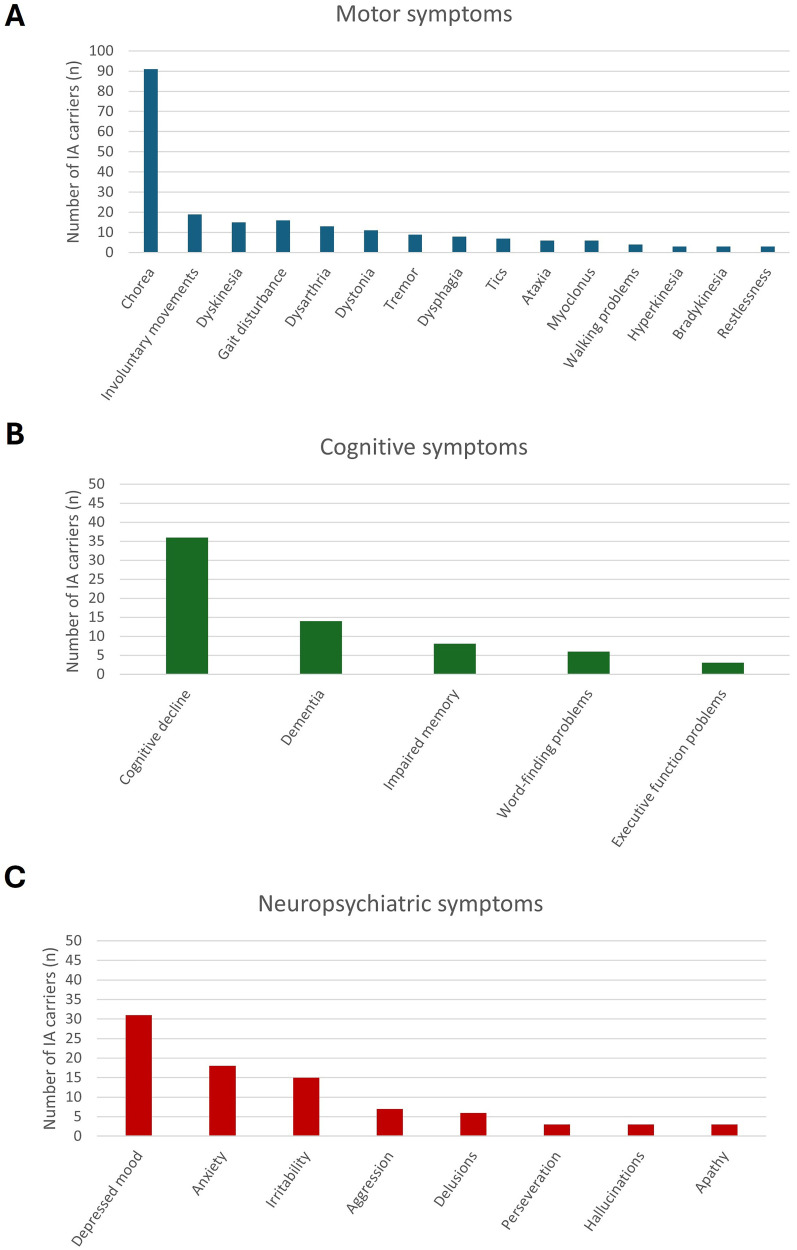
Bar charts of the most commonly reported symptoms in IA carriers (n = 189). A) The most commonly reported motor symptoms. B) The most commonly reported cognitive symptoms. C) The most commonly reported neuropsychiatric symptoms.

Motor, cognitive, and neuropsychiatric symptoms were identified in 151 (79.9%), 58 (30.7%), and 70 (37.0%) IA carriers, respectively. It is important to notice that part of these IA carriers were extracted from articles that focused on IA carriers with symptoms. Age at symptom onset was known in 120 IA carriers, with a mean age at onset of 57.2 years (SD 19.0, range: 4–87 years). Age at symptom onset was not significantly correlated with the CAG repeat length (R^2^ = 0.000, p-value = 0.873) [Figure 4]. Figure 4 reports the age at onset of the first reported symptom, regardless of its nature or potential relation to HD. Patients often presented with a combination of symptoms from multiple domains, and in many cases symptom onset was reported without indication of the specific domain (motor, cognitive, and neuropsychiatric). Figure 5 shows the most frequently reported motor (A), cognitive (B), and neuropsychiatric (C) symptoms. Only symptoms that were reported more than twice are shown. We did not differentiate between mild and severe symptoms, for example mild and severe chorea were subsumed under the category “chorea”.

##### Motor Symptoms

The most frequently reported motor symptoms were chorea, involuntary movements, dyskinesia, and gait disturbance [Figure 5(A)]. Some of these terms have overlapping meanings, but they are presented here as they are reported in the article. Chorea and involuntary movements were reported in 91, and 19 IA carriers, respectively. Of these 19 IA carriers with involuntary movements, six were also reported to have chorea. The location of the chorea or involuntary movements was reported in 25 IA carriers with chorea, and six IA carriers with involuntary movements. Eleven IA carriers were reported to have generalized chorea. Dyskinesia was reported in 15 IA carriers, including four IA carriers with medication-induced dyskinesia.^16,23^ The UHDRS Total Motor Score (TMS) was reported for five IA carriers, with an age range from 68 to 82 years, and their longer CAG repeat length ranging from 29 to 34. Their TMS scores were: 19, 22, 31, 39, and 72 (TMS ranges from 0 to 124, with higher scores indicating more severe motor impairment^69,70^).^26,31,32,38^

##### Cognitive Symptoms

Cognitive decline was reported in 36 IA carriers. The second most reported symptom was dementia (n = 14). Mini Mental State Examination (MMSE) scores were reported for six IA carriers, with an average of 25.0 (SD = 6.0, MMSE ranges from 0 to 30, with cognitively healthy people generally scoring 29 or 30^71^). Additionally, a Montreal Cognitive Assessment (MoCA) score was reported for three IA carriers, with an average of 21.7 (SD = 4.2, MoCA ranges from 0 to 30, in which a score of 26 or higher is considered normal^72^). Figure 5(B) shows the most frequently reported cognitive symptoms in IA carriers.

##### Neuropsychiatric symptoms

All reported neuropsychiatric symptoms were regrouped into the 11 symptom domains from the PBA-s, as described in the methods section. Six IA carriers had behavioral changes not further specified. Depressed mood (n = 31) was the most described neuropsychiatric symptom. IA carriers that were reported to have depression, affective symptoms, and lability were grouped as depressed mood. The second and third most reported neuropsychiatric symptoms were anxiety (n = 18), and irritability (n = 15), respectively. The IA carriers with psychosis (n = 4) and delusions (n = 2) were grouped under delusions. Another neuropsychological symptom of HD is apathy, which was reported in three IA carriers.^21,25^ However, loss of interest (n = 1), loss of motivation (n = 1), and self-neglect (n = 1) were reported, symptoms that might indicate apathy.^15,32,43^ Figure 5(C) displays the most frequently presenting neuropsychiatric symptoms in IA carriers.

A total of 12 from the 70 IA carriers with neurpsychiatric symptoms was diagnosed with the following psychiatric diagnoses: schizophrenia (n = 4),^21,25,33^ attention deficit disorder (ADD) or attention hyperactivity deficit disorder (ADHD, n = 2),^16,38^ obsessive compulsive disorder (OCD, n = 2),^16,21^ bipolar disorder (n = 2),^15,38^ and borderline disorder (n = 2).^21,25^ Additionally, eight IA carriers were diagnosed with an unspecified psychiatric disease or disorder,^21,27^ and two with a behavioral disorder.^21,40^ These 20 IA carriers with psychiatric diagnoses were not excluded from the symptom analysis, since these diagnoses do not have a genetic cause, and are mostly descriptive diagnoses.

#### Family history

Family history was documented in 89 IA carriers, of whom 29 IA carriers had a positive family history for HD, 48 IA carriers had a negative family history, and for 12 IA carriers the family history was unknown or not available. Among IA carriers with a positive family history, 14 (48.3%) had motor symptoms, whereas 44 (91.7%) of the IA carriers with a negative family history had motor symptoms. Additionally, for seven IA carriers it was reported that they transmitted an extended repeat (> 35 CAGs) to their offspring.

#### Medication

Some symptoms, such as chorea, can be induced by medication. Medication use was documented for 17 of 189 IA carriers, from which 12 IA carriers used medication [Supplemental Table 5]. The majority of medication reported was medication that is also used in the current clinical context for treating symptoms of HD, including antipsychotics (olanzapine, risperidone, haloperidol, sulpiride, prochlorperazine), antidepressants (amitriptyline, paroxetine, citalopram), and specific HD-related medication against chorea (tetrabenazine, tiapride). Additionally, medication often prescribed in the context of Parkinson's disease (PD, levodopa, carbidopa) was reported in two IA carriers who were not reported to have PD, but either a hypokinetic rigid syndrome with good effect of levodopa, or multiple motor symptoms, including restlessness, and tremors.^16,32^

### Cohort studies

Twenty-seven articles analyzed cohorts which included IA carriers.^16,21–24^^,47–68^ Nine articles specifically described the phenotype of IA carriers in their cohort.^16,21,23,51–54^^,57,64^ Additionally, six articles investigated an HD cohort, in which IA carriers were included.^22,47,48,50,59,65^ Table 3 summarizes details on these cohorts.

**Table 3.** IA carriers in IA phenotyping cohorts and HD cohorts.

Article	Cohort name	Inclusion: testing route	Inclusion period	Location	Non-expanded (n)	IA carriers (n) (IA ratio*)	Age IA carriers (mean ± SD) (range)	Symptomatic/ asymptomatic (n)
IA phenotyping cohorts								
Cubo, 2016^51^	European HD- registry	NR	1998–2014	European	657	76 (11.6%)	45.6 ± 15.6	NR/NR
Downing, 2016^53^	PREDICT-HD	Predictive	NR	US, Canada, Australia	301	21 (7.0%)	47.3 ± 10.4 (24.2–70.0)	NR/NR
Killoran, 2013^57^	PHAROS	Research	1999–2004	North America	637	50 (7.8%)	41.5 ± 7.0	NR/NR
Ha, 2012 and Dorsey, 2012^52,54^	COHORT	Mixed	2006–2009	US, Canada, Australia	695	50 (7.2%)	49.1 ± 13.1	0/50
Panegyres, 2015^64^	COHORT	Mixed	2006–2011	US, Canada, Australia	-	62	NR	NR/NR
Oosterloo, 2015^16^	–	Diagnostic	2001–2012	Netherlands	799	60 (7.5%)	58.8 ± 18.2 (5–86)	58**/0
Ramirez-Garcia, 2022^23^	–	Mixed	1994–2019	Mexico	-	34	42.4 ± 19.3 (12–74)	21/13
Ruiz de Sabando, 2024^21^	–	Mixed	NR	Spain	-	191	NR	78/NR
HD cohorts								
Alonso, 1997^47^	–	Research	NR	Mexico	-	8	NR	0/8
Brinkman, 1997^50^	–	Mixed	1984–1997	Canada	-	86	NR (6–93)	0/86
Raskin, 2000^65^	–	Mixed	NR	Brazil	-	8	NR	0/8
Maat-Kieviet, 2001^59^	–	Mixed	1993–1998	The Netherlands	-	26	NR	0/26
Despotov, 2021^22^	–	Mixed	1998–2018	Hungary	62	13 (21%)	NR	8/5
Barron, 1993^48^	–	Mixed	NR	Scotland	229	NR	NR	2/NR

*Ratio of IA carriers as a percentage of the non-expanded group of the cohort (CAG < 36). **From two IA carriers clinical data could not be obtained. NR = not reported, HD = Huntington's disease, HGECs = huntingtin gene expansion carriers (CAG ≥ 36).

Furthermore, three articles described a cohort consisting of patients with chorea or involuntary movements and reported IA carriers.^58,67,68^ Lastly, nine cohorts consisted of otherwise defined subjects, but identified IA carriers in their cohort.^24,49,55,56,60–63^^,66^ Table 4 shows the prevalence of IA carriers in these 12 cohorts.

**Table 4.** Prevalence of IA carriers in chorea cohorts and involuntary movement cohorts, and cohorts of other clinical conditions.

Article	Cohort type	Cohort size (n)	IA in cohort (n)	Prevalence in cohort	Main findings
Chorea and involuntary movements					
Vazquez-Mojena, 2013^68^	Chorea	130	NR	0%	IA carriers not reported in chorea cohort.
	Controls	63	5	7.9%	
Kim, 2024^58^	Chorea	954	11	1.2%	In older (> 55 years) participants, IAs were found more often in participants who had chorea compared to controls.
	Controls	941	13	1.4%	
Shan, 1997^67^	Involuntary movements	103	2	1.9%	No further data.
Cognitive decline and dementia					
Bessi, 2021^49^	Total cohort	75	5	6.7%	No difference in prevalence of IA carriers.
	SCD	46	3	6.5%	
	MCI	29	2	6.9%	
Mazzeo, 2022^60^	SCD	106	11	10.4%	No difference between SCD patients with and without an IA.
Moschini, 2022^63^	SCD	54	6	11.1%	No difference between SCD patients with and without an IA.
Ingannato, 2022^55^	Centenarians	143	16	11.2%	A higher prevalence of IA carriers in centenarians, suggesting a neuroprotective effect.
	Cognitive decline	232	12	5.4%	
	Controls	104	6	5.8%	
Neurodegenerative disorders					
Menédez-González, 2019^62^	AD	1126	68	6.0%	The AD group had a significantly higher prevalence of IA carriers compared to controls.
	PD	610	22	3.5%	
	FTLD	225	12	5.3%	
	Controls	509	15	2.9%	
Rosas, 2020^66^	FTD	440	30	6.8%	A higher frequency of IAs in patients with FTD (non-significant) and PNFA (significant), compared to controls.
	PNFA	59	8	13.6%	
	SD	32	1	3.1%	
	Controls	509	15	2.9%	
Pérez-Oliveira, 2022^24^	PD dementia	129	5	3.9%	None of the patient groups had a significant difference in frequency of IA carriers compared with the control group.
	PD	1010	42	4.2%	
	MSA	114	10	8.8%	
	DLB	354	20	5.6%	
	Controls	1039	41	3.9%	
McNicoll, 2008^61^	PD	484	25	5.2%	No relation between the CAG repeat length and the age at onset in PD was found in this cohort.
Ingannato, 2021^56^	ALS	106	8	7.5%	No significant association between the presence of IA and age at onset.

NR = not reported, SCD = subjective cognitive decline, MIC = mild cognitive impairment, AD = Alzheimer's disease, PD = Parkinson's disease, FTLD = frontotemporal lobar degeneration, FTD = frontotemporal dementia, PNFA = progressive nonfluent aphasia, SD = semantic dementia, MSA = multisystem atrophy, DLB = dementia with Lewy bodies, ALS = amyotrophic lateral sclerosis.

#### IA phenotyping cohorts

Nine articles specifically aimed at phenotyping IA carriers in their cohort [Table 3].^16,21,23,51–54^^,57,64^ All studies used the UHDRS to investigate motor, cognitive, behavioral, and functional changes.^70^

Cubo et al. analyzed data from the European HD-Registry, and described the clinical features of included participants with IAs. Longitudinal data were collected from 657 non-expanded participants (CAG < 36, age: 18–91 years), of whom 76 were IA carriers.^51^ At baseline, IA carriers were similar to healthy controls (HCs, CAG < 27) on all UHDRS subscales, PBA and demographics. One-year follow-up from 18 IA carriers and 39 paired sex-age HCs showed that IA carriers had significantly greater cognitive decline compared to HCs. In a subselection of older participants (age ≥ 60), IA carriers had significantly worse motor scores (median = 13.0, n = 10) compared to HCs (median = 2.0, n = 46) at baseline.

Downing et al. reported 21 IA carriers within the PREDICT-HD study. The study included 1078 HGECs, and 301 non-expanded participants, who previously underwent predictive testing.^53^ As part of the exclusion criteria participants must not have sufficient motor signs for a clinical HD diagnosis at study entry, no central nervous system diseases, and no clinical evidence of unstable medical or psychiatric illness. The authors did not find significant baseline or longitudinal differences between IA carriers and HCs.

In the Prospective Huntington At Risk Observational Study (PHAROS), Killoran et al. reported 50 IA carriers. PHAROS enrolled clinically unaffected participants, who had an affected parent or sibling (50–50 risk for HD-repeat expansion), but had chosen not to undergo predictive DNA testing, and determined their CAG repeat length as part of the PHAROS study.^57^ Individuals with severe depression or psychosis were excluded. The IA carriers (n = 50) were found to be similar to the HCs (n = 587) with regard to UHDRS measures of motor, cognitive, and general functioning. IA carriers had worse baseline scores on most behavioral features (7/11 PBA domains), although not significant. The IA carriers only scored significantly worse on the domains of apathy and suicidal thoughts.

The COHORT study included individuals that were clinically affected by HD, or HGECs, individuals from HD-families who had not undergone genetic testing, spouses, caregivers, and non-expanded participants. Both Ha et al. and Dorsey et al. described the COHORT study.^52,54^ In the 1985 subjects with a known CAG repeat length, 50 IA carriers were reported. None of them had a clinical HD diagnosis.^52^ Ha et al. compared the IA carriers (n = 50) to the HCs (n = 645). Subtle motor and cognitive abnormalities were found in the IA group, including a significantly higher total maximal dystonia score (part of the UHDRS) and lower scores on the Stroop word reading. Moreover, a significantly greater proportion of subjects in the IA group reported at least one suicide attempt, and a lower proportion of subjects in the IA group scored within the normal range on related parts of the behavioral domain of the UHDRS.^54^

Another study by Panegyres et al. also used data from the COHORT study, but included 12 additional IA carriers.^64^ They found that the behavioral frequency severity score, total functional assessment, independence scale, total functional capacity and MMSE did not significantly vary over the course of 4 years in the IA group (n = 62). Additionally, none of the IA carriers converted to manifest HD in those 4 years.

In a Dutch cohort of 1690 patients who underwent diagnostic testing for HD, 60 IA carriers were identified.^16^ Clinical data could be obtained from 58 of these IA carriers, of which 31 IA carriers had no positive family history for HD, ten had a positive family history, and for the other 17 the family history remained unclear. One IA carrier had clinical features suggesting HD, according to the author's in- and exclusion criteria. This patient had already been published by Groen et al.^32^ The data of the 58 IA carriers were described separately and were therefore also included in the case analysis in our review [Figure 3].

Ramirez-Garcia et al. detected 34 IA carriers in a cohort of 1893 individuals who had previously undergone testing due to suspected HD, presymptomatic counseling, or for research purposes.^23^ The route of testing was not reported individually for the IA carriers. Two of the 15 IA carriers with a positive family history for HD showed symptoms, while all 19 IA carriers with a negative family history showed symptoms. These 19 IA carriers presented with motor disturbances, of whom 17 manifested involuntary movements or chorea. All IA carriers were described separately, and were therefore also included in the case analysis in our review [Figure 3].

Ruiz de Sabando et al. included 191 IA carriers in their study.^21^ IA carriers were identified among subjects with signs compatible with HD, and their family members that were referred for genetic testing and counselling, and individuals from the Spanish general population. They reported that somatic expansions were CAG-repeat- and age-dependent in IA carriers (n = 135). Symptomatic IA carriers (n = 78) did not show increased somatic CAG expansions or CAG-dependent symptom onset compared to IA carriers from the general population (n = 46). Symptomatic IA carriers presented with motor (85%), cognitive (27%), and/or behavioral (29%) signs. The average age of onset was 58.7 years ± 18.6 years (n = 49, range: 22–88 years). Clinical onset in IA carriers did not show a CAG-dependent correlation. Clinical data of a subset of IA carriers (n = 89) were reported, but their recruitment background was not specified. Additionally, four IA carriers from a HD-family were described. These 93 IA carriers were included in the case analysis in our review [Figure 3].

### IA carriers in HD cohorts

We found six articles describing HD cohorts that reported IA carriers.^22,47,48,50,59,65^ In four of these cohorts, all IA carriers were included in the control group (CAG < 36) and were without symptoms [Table 3].^47,50,59,65^ Alonso et al. initially reported one IA carrier, based on the IA range at that time (30–35 CAGs, in 1997).^47^ Applying the current range, a total of eight IA carriers would now be identified, all of whom were included as controls (n = 96) in their cohort.^47^ Another cohort by Brinkman et al. only included individuals with a CAG > 28 as their longer allele.^50^ They included individuals that were asymptomatic and at-risk for HD as their control group (n = 321) and reported 86 (26.8%) IA carriers in this group.

The two other cohorts reported IA carriers with symptoms. Barron et al. reported two IA carriers, with 31 and 33 CAG repeats, as “affected”, with no further description.^48^ Additionally, Despotov et al. identified 13 IA carriers, of whom eight were symptomatic.^22^ These eight symptomatic IA carriers were all tested for diagnostic purposes. They presented with HD-like symptoms, such as chorea, perioral dyskinesia, and cognitive decline. Three of them presented with additional symptoms, such as ataxia, dysphagia, dysarthria, and convulsions. For one of these cases there was evidence for an alternative diagnosis (alcohol and medication abuse). Only one of these symptomatic IA carriers had a positive family history for HD; this case was separately described and therefore included in the case analysis in our review [Figure 3].^22^

#### IA carriers in chorea cohorts and involuntary movement cohorts

Vázquiz-Mojena et al. investigated a cohort of 130 patients exhibiting chorea (n = 130), and a control group without chorea (n = 63)[Table 4].^68^ They identified five IA carriers in their control group. None of the IA carriers had a family history of HD, or clinical signs suggestive of HD or other neurological disorders. For the chorea group, the authors only reported pathological CAG repeat lengths. Consequently, it is unknown whether individuals with IA were present among the patients exhibiting chorea.^68^

In another cohort of chorea patients (n = 954) by Kim et al., 11 (1.2%) IA carriers were reported, with CAG repeat lengths ranging between 27–30 CAG repeats [Table 4].^58^ In the control group without chorea (n = 941), 13 (1.4%) IA carriers were reported, with a similar CAG repeat range (CAG = 27–31). Additionally, 213 patients with chorea were diagnosed with HD (CAG > 39). In a subanalysis in 871 participants aged over 55 years, of whom 381 had chorea, IA alleles were found in nine (2.4%) patients with chorea and in three (0.6%) control individuals. The authors therefore reported that IAs were significantly associated with a higher risk for chorea in participants of 55 years and older.^58^

In addition, Shan et al. reported two IA carriers in a group of 103 Chinese patients with involuntary movements who were tested for HD [Table 4]. These two patients with stereotypic movements showed a longer allele of 29 and 30 repeats.^67^ No further data on these IA carriers was reported.

#### The influence of IAs on other clinical conditions

Besides the clinical phenotype of IA carriers, the effect of IAs on other diseases was studied in several cohorts. There were nine articles describing cohorts consisting of patients with a range of different clinical conditions [Table 4]. The results of these articles were ambiguous. Some results suggested a possible neuroprotective effect of IAs.^55,56^ For example, Ingannato et al. reported a higher prevalence of IA carriers in centenarians.^55^ They also reported two patients with amyotrophic lateral sclerosis (ALS) who carried IAs and had a non-significantly lower plasma neurofilament light chain (NfL) concentration, compared to non-IA carriers.^56^ In contrast, the prevalence of IA carriers was reported to be significantly higher in several disease cohorts, including frontotemporal dementia (FTD), progressive nonfluent aphasia (PNFA), and AD, compared to controls. Indicating that IAs may contribute to an increased risk of developing these conditions.^62,66^

Separate data were provided for 10 IA carriers in the cohort of Pérez-Oliveira et al., who were therefore included in the case analysis in our review [Figure 3]. However, due to the absence of clinical data, they were excluded from the symptom analysis.

### Neuroimaging

#### Neuroimaging in cases

Neuroimaging was performed in 37 IA carriers (15.2% of 243 IA carriers), 28 of these were included in the symptom analysis in our review.^15,16,23,25,26,28,31–33^^,36,38,39,42,43^ The other nine were excluded, and therefore also excluded from our analysis of neuroimaging results [Table 2].^16,25,37,41,45^ Magnetic resonance imaging (MRI) was performed in 19 IA carriers.^15,16,23,25,31,32,36,38,39^ In eight IA carriers another neuroimaging technique, i.e., computed tomography (CT) or positron emission tomography (PET), was performed.^16,23,26,28,33,42,43^ Additionally, in one IA carrier both MRI and [11C]-raclopride PET-scan were performed.^32^

Atrophy was the most frequently reported finding, which was reported in 14 IA carriers (mean age: 63,7 ± 15,9 years, range: 30–82 years). Generalized atrophy was reported in four IA carriers.^23,31,32,38^ Furthermore, cortical (n = 5), subcortical (n = 3), caudate nucleus (n = 5), cerebellar (n = 1), and hippocampal atrophy (n = 1) were reported.^15,23,26,28,33,36,39^ The individuals with atrophy were reported to have motor symptoms (n = 13), cognitive symptoms (n = 6), and neuropsychological symptoms (n = 10). One IA carrier had enlargement of the lateral ventricles.^28^ Chorea was the most reported motor symptom in these individuals (n = 12). Additionally, of the five IA carriers with caudate nucleus atrophy, four were reported to have chorea. Moreover, two IA carriers with mild generalized atrophy were reported to have late-onset HD, and one IA carrier with cortical and cerebellar atrophy was reported to have HD.^31–33^

Apart from atrophy, white matter lesions (n = 2),^16^ small white matter changes in the putamen (n = 1),^15^ and iron deposits in the basal ganglia (n = 1)^25^ were reported. Three IA carriers were reported with ischemia, of which one in the caudate nucleus.^15,38,39^ Using PET scan, severe glucose hypometabolism in the caudate nucleus was found in another IA carrier, who was reported to have subtle oculomotor abnormalities, and slight arm incoordination.^42^

#### Neuroimaging in cohorts

Downing et al. reported brain MRI data from 21 IA carriers and 280 HCs (CAG < 27).^53^ Compared to HCs, IA carriers did not show a significantly different striatal volume decrease over time (11 years follow-up). However, IA carriers did have a slightly lower striatal volume, at baseline as well as at the follow-up moments.^53^

In the cohort of Ramirez-Garcia et al. MRI data was available from nine IA carriers, from which eight showed structural changes in the brain.^23^ Four of these eight IA carriers were included in the neuroimaging analysis in cases above. From the remaining five, no further data was reported, or they were excluded due to other diagnoses [Table 2, and Supplemental table 1].

## Discussion

To our knowledge, this is the first scoping review of the clinical phenotype of carriers of an IA of the *HTT* gene. We found that a significant proportion of the IA carriers reported in literature had symptoms. In the published IA carriers, motor, cognitive, and neuropsychiatric symptoms were identified in 79.9%, 30.7%, and 37.0% of the IA carriers, respectively. These also included symptoms that are often found in HD patients, such as chorea, which was reported in 60.3% of IA carriers with motor symptoms.^3,73^ However, symptoms such as cognitive decline, depression, and anxiety are not HD-specific, and common in the general population. Moreover, these symptoms could potentially be explained by other disorders, such as Alzheimer's disease.^74–76^ Additionally, it is not reported whether a clinician determined the symptoms in these IA carriers. It is also important to notice that a proportion of the IA carriers in our case analysis were tested for diagnostic purposes and were therefore by definition symptomatic.^16,21^

A considerable proportion (22 out of 28) of IA carriers was reported to have abnormalities in neuroimaging. Generalized atrophy and white matter lesions were reported in IA carriers, which are non-specific and common in the general older population.^77–80^ However, caudate atrophy was also reported in five IA carriers, which may be more specific for HD-related pathology.^81^ Moreover, four of these individuals presented with chorea, which is, as mentioned above, relatively specific for HD. Downing et al. uniquely compared IA carriers with HCs, providing more reliable data. They found no significant difference between IA carriers and HCs in striatal volume decline over 11 years, although IA carriers had slightly lower striatal volumes at baseline and follow-up.

The results of the published cohort studies vary regarding the clinical phenotype of IA carriers. Most cohorts showed no (significant) clinical motor, cognitive, or behavioral differences between IA carriers and HCs.^51,53,57,64^ Some cohorts did report non-significant impairments in IA carriers. For example, IA carriers were reported to have slightly worse scores on motor, cognitive, or behavioral measures, compared to HCs.^53,54,57^ Cubo et al. reported that IA carriers were similar to HCs at baseline, but had a statistically significantly greater cognitive decline compared to sex- and age-matched HCs at one year follow-up.^51^ Additionally, Ha et al. reported a significantly higher total maximal dystonia score in IA carriers compared to HCs.^54^ Other papers reported more neuropsychiatric symptoms in IA carriers compared to HCs. Cohorts of IA carriers scored significantly worse on suicidal thoughts and had significantly greater proportion of subjects that reported at least one suicide attempt, compared to HCs.^54,57^

### Current hypotheses

Multiple hypotheses exist regarding the clinical phenotype of IA carriers. According to one current hypothesis, IA carriers would have an HD onset that falls beyond their life expectancy, based on shorter repeat lengths leading to a later onset.^82^ Extrapolation of the model that describes the relationship between CAG repeat length and age of onset yields an age of onset for IA carriers of around 90–100 years, or even higher.^10,83,84^ Variation in age of onset among HD patients suggests similar variability in IA carriers. This assumption is supported by the broad range in age at symptom onset for IA carriers in our review. Due to this variability some IA carriers might be expected to show symptoms within their lifetime. In contrast, Ingannato et al. suggested that IAs might have a neuroprotective effect, and even play a positive role in longevity.^55^

Another hypothesis is based on CAG mosaicism, meaning that there are differences in the number of CAGs in cells and tissues of the same individual.^21^ Somatic expansions have been found in the brain of HD patients.^21,85,86^ The hypothesis suggests that the CAG repeat might expand into the recognized pathological range in the brain tissue of IA carriers. However, contradictory to this hypothesis, no difference in somatic CAG expansions between symptomatic IA carriers and population controls has been found in a study by Ruiz de Sabando et al.^21^

A further hypothesis is that a loss of interruption (LOI) in the *HTT* gene could lengthen the uninterrupted CAG repeat length. The CAG repeat in the *HTT* gene is followed immediately by an interrupting sequence (CAA-CAG). This CAA triplet can mutate into a CAG triplet, resulting in an uninterrupted CAG repeat that has two additional CAG triplets.^28,87^ These additional CAG triplets may shift individuals from the IA range to the reduced penetrance range. An LOI is not detected with the general fragment-based genetic test for HD.^28^ Additionally, the current genetic test comes with uncertainty; the exact number of CAG repeats may differ by one or two repeats depending on the laboratory.^88^ This implies that an individual with a reported repeat length of 35 CAGs, may have an actual repeat length of up to 37 CAGs.

These hypotheses are not mutually exclusive. Assuming that IA carriers would have a very late onset, both CAG mosaicism and LOI could contribute to an earlier age at onset. However, all three of these hypotheses require further investigation. More research on somatic CAG expansions and LOI in IA carriers is particularly needed.

### Limitations

Researching literature on IA carriers entails several challenges, starting with the definition of IA. IAs are defined as being below the affected CAG range, but having the potential to expand into the disease range (CAG > 35) within one generation.^16^ The range of IAs has been adjusted whenever a lower repeat length has been found to expand into the disease range within one generation. Therefore, data of the smaller repeat lengths (27–30 CAG) is often missing in older articles.

Additionally, the classification of IAs is based solely on their capacity to expand into the disease range within one generation (intergenerational instability). However, the intergenerational instability of the CAG repeat is not necessarily related to the development of pathological processes in an individual. Therefore, the range associated with the development of a clinical phenotype may differ from the range defined by the potential to expand into the disease range within one generation.

Another challenge is the wide variety in nomenclature used for the 27–35 CAG range. Currently, the widely acknowledged term is Intermediate Alleles (IAs), but other names that have been used are ‘high normal alleles’, ‘large normal’, ‘mutable normal alleles’, ‘borderline alleles’, ‘grey area’, and ‘premutation alleles’.^21,38,89^ In addition, the term ‘Reduced Penetrance (RP)’ was used in literature, which is currently reserved for the 36–39 CAG range.^16^ To overcome this limitation, our search strategy was set up as broad as possible, by also including the concept ‘CAG’ with ‘27–35’ (and ‘27’, ‘28’, etc.). Despite our broad search strategy we might have missed some articles using nomenclatures other than those included in our search.

Furthermore, there appears to be a publication bias towards IA carriers with symptoms. There is a tendency to only publish case reports of symptomatic IA carriers. Conversely, if data of asymptomatic IA carriers are reported, they are often pooled with data from HCs. In this way data on asymptomatic IA carriers are harder to obtain or include in meta-analysis. The way data is reported makes it both harder to determine the actual prevalence of symptoms in IA carriers, and more difficult to identify potential differences between IA carriers and HCs.

There is also a selection bias regarding symptomatic or presymptomatic testing. IA carriers from HD families are often identified through testing in asymptomatic stages, while IA carriers from the general population are often tested as part of a diagnostic process, particular in relation to clinically ascertained chorea. Therefore, IA carriers found in the general population are more likely to have symptoms, which is underlined by earlier research. For example, Ramirez-Garcia et al. reported that 100% of IA carriers included from the general population had symptoms, while only 13.3% of the IA carriers from HD families had symptoms.^23^ Our review also demonstrated that motor symptoms were more prevalent among IA carriers with a negative family history (91.7%) compared to those with a positive family history (48.3%). The likely reason is that IA carriers with a negative family history were tested for diagnostic purposes. A similar selection bias is also reported for the reduced penetrance range (CAG = 36–39).^90^ Participants with an allele in the reduced penetrance range (RP carriers) in the study of Van der Zwaan et al. had an earlier onset than was to be expected from literature. The authors hypothesize that RP carriers with symptoms are more likely to participate in a study. Another issue with participant selection is that some cohorts excluded symptomatic patients, thereby affecting the proportion of IA carriers presenting with symptoms.

Lastly, there is a notable lack of information in the literature, making it difficult to contextualize results. For instance, cohort studies did often not report how participants were selected, or how IA carriers were identified. Moreover, detailed symptom descriptions or differential diagnosis analyses were missing for a large number of IA carriers. For example, several IA carriers diagnosed with PD or Parkinsonism lacked information about the response to levodopa. Some of these IA carriers might have been misdiagnosed as PD although they showed symptoms compatible with HD.^91^

## Conclusion

Based on this review we argue that there is not enough evidence to draw a clear conclusion on the clinical phenotype of individuals carrying an intermediate allele of the *HTT* gene. Most of the symptoms and neuroimaging abnormalities reported in IA carriers (CAG 27–35) are commonly found in the general population. Furthermore, both publication bias and selection bias play a substantial role in the published evidence on IA carriers. These and other challenges discussed above make it hard to get a clear picture of the clinical phenotype of IA carriers from literature. Future studies should therefore focus on reporting separate, more detailed clinical and biological data on IA carriers, from larger cohorts of IA carriers, age- and sex-matched with healthy controls (< 27 CAG). More research is needed to provide a better insight into the clinical phenotype of IA carriers, both to improve prognosis and counseling for IA carriers, and to enhance our understanding of the pathogenic processes caused by the CAG expansion in the *HTT* gene.

## Supplemental Material

sj-pdf-1-hun-10.1177_18796397251397683 - Supplemental material for The clinical phenotype of carriers of intermediate alleles in the huntingtin gene: A scoping review Supplemental material, sj-pdf-1-hun-10.1177_18796397251397683 for The clinical phenotype of carriers of intermediate alleles in the huntingtin gene: A scoping review by Anna van Hofslot, Mayke Oosterloo, Joost J.A. de Jong, Ruben L. Andriessen, Susanne T. de Bot and David E. J. Linden in Journal of Huntington's Disease
