# Supplementary material for: The clinical phenotype of carriers of intermediate alleles in the huntingtin gene: A scoping review
Source: J Huntingtons Dis. 2025 Dec 17;15(1):3–19. doi: 10.1177/18796397251397683 (PMC12847463; doi:10.1177/18796397251397683)
Supplement: sj-pdf-1-hun-10.1177_18796397251397683 - Supplemental material for The clinical phenotype of carriers of intermediate alleles in the huntingtin gene: A scoping review [file sj-pdf-1-hun-10.1177_18796397251397683.pdf]

Search strategy for PubMed, Web of Science, and EMBASE

July 2024

Search strategy in PubMed: (n= 340 hits)	
Concept #1	huntington disease[MeSH Terms] OR "Huntingt*"[Title/Abstract] OR HTT[Title/Abstract] OR IT15[Title/Abstract] OR "interesting transcript 15"[Title/Abstract:~0] OR "important transcript 15"[Title/Abstract:~0]
Concept #2	"intermediate allele*"[Title/Abstract] OR "intermediate repeat*"[Title/Abstract] OR "intermediate allele"[Title/Abstract:~4] OR "intermediate repeat"[Title/Abstract:~4] OR "intermediate alleles"[Title/Abstract:~4] OR "intermediate repeats"[Title/Abstract:~4] OR "intermediate CAG"[Title/Abstract:~4] OR "intermediate range"[Title/Abstract:~4] OR "intermediate size"[Title/Abstract:~4] OR "intermediate size*"[Title/Abstract] OR "intermediate length"[Title/Abstract:~4] OR "intermediate expansion"[Title/Abstract:~4] OR "intermediate expansion*"[Title/Abstract] OR "intermediate genotype"[Title/Abstract:~4] OR "IA"[Title/Abstract] OR "IAs"[Title/Abstract] OR "large normal"[Title/Abstract] OR "mutable normal"[Title/Abstract:~0] OR "27-35 repeat"[Title/Abstract:~3] OR "27-35 repeats"[Title/Abstract:~3] OR "27-35 allele"[Title/Abstract:~3] OR "27-35 alleles"[Title/Abstract:~3] OR "27-35 range"[Title/Abstract:~3] OR "27-35 CAG"[Title/Abstract:~3] OR "27 repeat"[Title/Abstract:~3] OR "28 repeat"[Title/Abstract:~3] OR "29 repeat"[Title/Abstract:~3] OR "30 repeat"[Title/Abstract:~3] OR "31 repeat"[Title/Abstract:~3] OR "32 repeat"[Title/Abstract:~3] OR "33 repeat"[Title/Abstract:~3] OR "34 repeat"[Title/Abstract:~3] OR "35 repeat"[Title/Abstract:~3] OR "27 repeats"[Title/Abstract:~3] OR "28 repeats"[Title/Abstract:~3] OR "29 repeats"[Title/Abstract:~3] OR "30 repeats"[Title/Abstract:~3] OR "31 repeats"[Title/Abstract:~3] OR "32 repeats"[Title/Abstract:~3] OR "33 repeats"[Title/Abstract:~3] OR "34 repeats"[Title/Abstract:~3] OR "35 repeats"[Title/Abstract:~3] OR "27 allele"[Title/Abstract:~3] OR "28 allele"[Title/Abstract:~3] OR "29 allele"[Title/Abstract:~3] OR "30 allele"[Title/Abstract:~3] OR "31 allele"[Title/Abstract:~3] OR "32 allele"[Title/Abstract:~3] OR "33 allele"[Title/Abstract:~3] OR "34 allele"[Title/Abstract:~3] OR "35 allele"[Title/Abstract:~3] OR "27 alleles"[Title/Abstract:~3] OR "28 alleles"[Title/Abstract:~3] OR "29 alleles"[Title/Abstract:~3] OR "30 alleles"[Title/Abstract:~3] OR "31 alleles"[Title/Abstract:~3] OR "32 alleles"[Title/Abstract:~3] OR "33 alleles"[Title/Abstract:~3] OR "34 alleles"[Title/Abstract:~3] OR "35 alleles"[Title/Abstract:~3] OR "27 CAG"[Title/Abstract:~3] OR "28 CAG"[Title/Abstract:~3] OR "29 CAG"[Title/Abstract:~3] OR "30 CAG"[Title/Abstract:~3] OR "31 CAG"[Title/Abstract:~3] OR "32 CAG"[Title/Abstract:~3] OR "33 CAG"[Title/Abstract:~3] OR "34 CAG"[Title/Abstract:~3] OR "35 CAG"[Title/Abstract:~3]
Search Strategy	**#1 AND #2**<br><br>(huntington disease[MeSH Terms] OR "Huntingt*"[Title/Abstract] OR HTT[Title/Abstract] OR IT15[Title/Abstract] OR "interesting transcript 15"[Title/Abstract:~0] OR "important transcript 15"[Title/Abstract:~0])<br><br>AND<br><br>("intermediate allele*"[Title/Abstract] OR "intermediate repeat*"[Title/Abstract] OR "intermediate allele"[Title/Abstract:~4] OR "intermediate repeat"[Title/Abstract:~4] OR "intermediate alleles"[Title/Abstract:~4] OR "intermediate repeats"[Title/Abstract:~4] OR "intermediate CAG"[Title/Abstract:~4] OR "intermediate range"[Title/Abstract:~4] OR

	"intermediate size"[Title/Abstract:~4] OR "intermediate size*"[Title/Abstract] OR "intermediate length"[Title/Abstract:~4] OR "intermediate expansion"[Title/Abstract:~4] OR "intermediate expansion*"[Title/Abstract] OR "intermediate genotype"[Title/Abstract:~4] OR "IA"[Title/Abstract] OR "IAs"[Title/Abstract] OR "large normal"[Title/Abstract] OR "mutable normal"[Title/Abstract:~0] OR "27-35 repeat"[Title/Abstract:~3] OR "27-35 repeats"[Title/Abstract:~3] OR "27-35 allele"[Title/Abstract:~3] OR "27-35 alleles"[Title/Abstract:~3] OR "27-35 range"[Title/Abstract:~3] OR "27-35 CAG"[Title/Abstract:~3] OR "27 repeat"[Title/Abstract:~3] OR "28 repeat"[Title/Abstract:~3] OR "29 repeat"[Title/Abstract:~3] OR "30 repeat"[Title/Abstract:~3] OR "31 repeat"[Title/Abstract:~3] OR "32 repeat"[Title/Abstract:~3] OR "33 repeat"[Title/Abstract:~3] OR "34 repeat"[Title/Abstract:~3] OR "35 repeat"[Title/Abstract:~3] OR "27 repeats"[Title/Abstract:~3] OR "28 repeats"[Title/Abstract:~3] OR "29 repeats"[Title/Abstract:~3] OR "30 repeats"[Title/Abstract:~3] OR "31 repeats"[Title/Abstract:~3] OR "32 repeats"[Title/Abstract:~3] OR "33 repeats"[Title/Abstract:~3] OR "34 repeats"[Title/Abstract:~3] OR "35 repeats"[Title/Abstract:~3] OR "27 allele"[Title/Abstract:~3] OR "28 allele"[Title/Abstract:~3] OR "29 allele"[Title/Abstract:~3] OR "30 allele"[Title/Abstract:~3] OR "31 allele"[Title/Abstract:~3] OR "32 allele"[Title/Abstract:~3] OR "33 allele"[Title/Abstract:~3] OR "34 allele"[Title/Abstract:~3] OR "35 allele"[Title/Abstract:~3] OR "27 alleles"[Title/Abstract:~3] OR "28 alleles"[Title/Abstract:~3] OR "29 alleles"[Title/Abstract:~3] OR "30 alleles"[Title/Abstract:~3] OR "31 alleles"[Title/Abstract:~3] OR "32 alleles"[Title/Abstract:~3] OR "33 alleles"[Title/Abstract:~3] OR "34 alleles"[Title/Abstract:~3] OR "35 alleles"[Title/Abstract:~3] OR "27 CAG"[Title/Abstract:~3] OR "28 CAG"[Title/Abstract:~3] OR "29 CAG"[Title/Abstract:~3] OR "30 CAG"[Title/Abstract:~3] OR "31 CAG"[Title/Abstract:~3] OR "32 CAG"[Title/Abstract:~3] OR "33 CAG"[Title/Abstract:~3] OR "34 CAG"[Title/Abstract:~3] OR "35 CAG"[Title/Abstract:~3])

Search strategy in Web of Science: (n= 436 hits)	
Concept #1	TS=(Huntingt* OR HTT OR IT15 OR "interesting transcript 15" OR "important transcript 15")
Concept #2	TS=(((intermediate) NEAR/4 (allele* OR repeat* OR CAG OR range OR size* OR length OR expansion* OR genotype)) OR IA OR IAs OR "large normal" OR "mutable normal" OR (("27-35" OR 27 OR 28 OR 29 OR 30 OR 31 OR 32 OR 33 OR 34 OR 35) NEAR/3 (allele* or repeat* or CAG)))
Search Strategy	**#1 AND #2**<br>(TS=(Huntingt* OR HTT OR IT15 OR "interesting transcript 15" OR "important transcript 15"))<br>AND<br>(TS=(((intermediate) NEAR/4 (allele* OR repeat* OR CAG OR range OR size* OR length OR expansion* OR genotype)) OR IA OR IAs OR "large normal" OR "mutable normal" OR (("27-35" OR 27 OR 28 OR 29 OR 30 OR 31 OR 32 OR 33 OR 34 OR 35) NEAR/3 (allele* or repeat* or CAG))))

Web of Science has no controlled vocabulary.

Search strategy in Embase: (n= 479 hits)	
Concept #1	(Huntingt* or HTT or IT15 or "interesting transcript 15" or "important transcript 15").ti,ab,kw. or exp Huntington chorea/)
Concept #2	((intermediate adj4 (allele* or repeat* or CAG or range or size* or length or expansion* or genotype)) or IA or IAs or "large normal" or "mutable normal" or (("27-35" or "27" or "28" or "29" or "30" or "31" or "32" or "33" or "34" or "35") adj3 (repeat* or allele* or CAG))).ti,ab,kw.
Search Strategy	**#1 AND #2**<br>(((Huntingt* or HTT or IT15 or "interesting transcript 15" or "important transcript 15").ti,ab,kw.) or exp Huntington chorea/)<br>AND<br>(((intermediate adj4 (allele* or repeat* or CAG or range or size* or length or expansion* or genotype)) or IA or IAs or "large normal" or "mutable normal" or (("27-35" or "27" or "28" or "29" or "30" or "31" or "32" or "33" or "34" or "35") adj3 (repeat* or allele* or CAG))).ti,ab,kw.)
